# Staining susceptibility and the effect of different stain removal techniques on the optical properties of injectable composite resins

**DOI:** 10.3389/froh.2025.1556155

**Published:** 2025-02-28

**Authors:** Nancy Soliman Farghal, Fadia Awadalkreem, Shahistha Parveen Dasnadi, Shatha Habush, Nur Ali Hatab, Asmaa Harhash

**Affiliations:** ^1^ Department of Restorative Dentistry and Endodontics, RAK College of Dental Sciences, RAK Medical and Health Sciences University, Ras Al-Khaimah, United Arab Emirates; ^2^ Department of Dental Biomaterials, Faculty of Dentistry, Tanta University, Tanta, Egypt; ^3^ Department of Prosthodontics, RAK College of Dental Sciences, RAK Medical and Health Sciences University, Ras Al-Khaimah, United Arab Emirates; ^4^ Department of Orthodontics, RAK College of Dental Sciences, RAK Medical and Health Sciences University, Ras Al-Khaimah, United Arab Emirates; ^5^ Department of Oral Surgery, RAK College of Dental Sciences, RAK Medical and Health Sciences University, Ras Al-Khaimah, United Arab Emirates; ^6^ Restorative and Esthetic Dentistry Department, University of Fujairah, Fujairah, United Arab Emirates

**Keywords:** aesthetic, bleaching, color stability, gloss, injectable composite, nanofilled composite, optical properties, polishing

## Abstract

**Introduction:**

The injectable composite resin technique using highly filled flowable composite for anterior restorations is relatively new. This study aims to detect the staining susceptibility and the effect of polishing and bleaching agents and their combination on the stain removal and surface gloss of the injectable composite resins compared to sculptable nanofilled composite.

**Methods:**

Eighty-four disc-shaped specimens were prepared from two injectable composite resins: Beautifil Flow Plus X (BFP) and G-ænial Universal Injectable (GUI) and one sculptable nanofilled composite; Filtek™ Z350XT Universal Restorative (FUR), immersed in an instant coffee solution for 12 days. The specimens from each material were divided into four groups (*n* = 7) according to the stain-removal method: Group 1 (control): no stain removal treatment. Group 2: Polished with Super-Snap Buff Polisher and Direct DiaPaste for 60 s. Group 3: Bleached with Opalescence Boost 40% for one hour (3 rounds/20 min each). Group 4: bleached and polished. A Spectrophotometer recorded the color parameter initially (T_0_), after staining (T_1_) and after stain removal methods (T_2_) and color change (ΔE_00_) was calculated. Gloss (GU) was recorded initially and after stain removal methods using a glossmeter. Surface morphology was examined with Scanning Electron Microscopy. The data was analyzed using One and Two-way ANOVA and Tukey's HSD *post hoc* test using SPSS software at a 5% significance level.

**Results:**

All tested materials showed clinically unacceptable staining susceptibility after coffee immersion and stain removing methods (ΔE_00_ >1.8), with FUR exhibiting the highest change (26.2 ± 2.6). In-office bleaching and combined bleaching/polishing significantly reduced color change for FUR (*P* < 0.05), while all stain removal methods was equally effective for BPF and GUI (*P* > 0.05). Surface gloss remained unchanged with the highest values after staining and bleaching for all materials (52.8 ± 11.2–49.7 ± 9.4, *P* > 0.05) but significantly decreased after polishing alone or combined with bleaching (31.6 ± 5.7–15.4 ± 1.5, *P* < 0.05).

**Conclusion:**

Injectable composites exhibited lower staining susceptibility than the sculptable nanofilled composite. No stain-removing method restored the color for all composites to the clinically acceptable threshold. In-office bleaching with Opalescence Boost 40% effectively maintained optimal surface gloss, while polishing alone or after bleaching is not recommended due to its negative impact on gloss.

## 1 Introduction

The aesthetic and functional rehabilitation of anterior teeth is one of the main objectives of restorative dentistry that has rapidly evolved in recent years (1). Among the various restorative options, direct resin composite restorations present a versatile and less invasive alternative to ceramic restorations (2). They surpass indirect composite restorations regarding reduced laboratory time and costs (3). Although the conventional incremental layering technique has been the most widely recognized composite application method for direct anterior restorations among dental practitioners, it is considered quite time-consuming. Besides, the operator must carefully apply and cure each composite material layer to ensure proper adaptation and optimize the aesthetic outcome. Thus, the operator's precision, experience, and skill are critical factors in the success of this technique (4).

The recently introduced injectable composite technique has gained attention because of its simplicity, cost-effectiveness, and lesser demand on clinician expertise compared to direct and indirect restorative methods (5). This method completely restores the involved teeth by directly injecting the specially formulated injectable composite materials into a perforated transparent silicon index, giving a highly accurate final composite with predictable results (6). The success of such restorations relies on the free-hand injection molding technique as well as on the properties of the utilized injectable composites; these are highly filled flowable restorative materials with an innovative production process that allows modifications to the filler size and salinization mechanism. These modifications enable the injectable composites to have the improved adaptability and flow of the conventional flowable composites, with increased wear resistance, surface hardness, and flexure strength (7–9).

Nevertheless, the utilization of injectable resin composites for aesthetic anterior purposes is relatively new, and further research is required to understand its long-term performance fully. Previous case studies of this technique have reported discoloration after prolonged exposure to the oral environment (6, 10). The unacceptable color change of the restoration is considered an important concern causing patients dissatisfaction due to increased costs for restoration replacement.

Polishing and bleaching techniques employing commercially available at-home and in-office bleaching agents are utilized to eliminate the discoloration of teeth and composite restorations (11). The peroxide in the bleaching agent will break down into free radicals that penetrate the material and break down the pigmentation molecules, therefore eliminating or diminishing discoloration (12, 13). Surface polishing, on the other hand, depends on surface abrasion of the material's treated surface (14). Previous research studies have documented different color shift degrees in tooth-colored restorative materials due to either bleaching procedures or repolishing after exposure to staining beverages, with no preference for either method (15–18). Korać et al. and Alharbi et al. reported that bleaching is regarded as an effective approach for the elimination of surface stains in composite restorations (11, 19). While Korkut and Haciali determined in their investigation that the repolishing technique effectively restored the color of stained composite materials (20). Nevertheless, Rodrigues et al. reported that the color stability of resin composites subjected to staining agents is improved when they are repolished immediately following bleaching (21). In addition to their color stability, anterior restorations’ ability to acquire a smooth, glossy finish that resembles dental enamel and maintain this surface quality over time despite continual exposure to intraoral challenges as well as abrasives, such as toothbrushing and repolishing is another crucial aspect of their aesthetics (22, 23).

Since the stain removal method's efficacy relies on the stain's nature and the composition of the material being treated (18, 21, 24), it had to be determined if the same applies to the newly developed injectable resin restorative material. To the best of our understanding, no previous study has investigated the staining susceptibility of injectable composites. Therefore, this *in vitro* study aims to assess the staining susceptibility of the newly introduced injectable resin restorative materials compared to a conventional sculptable composite resin. Additionally, the study aims to compare and evaluate the effect of in-office bleaching and surface polishing techniques or their combination on removing stains and the surface gloss of the tested materials. The first null hypothesis states that there would be no difference in staining susceptibility between the tested materials, and the second null hypothesis states that neither the material type nor the stain removing method would influence the color or the surface gloss of the tested materials.

## 2 Materials and methods

### 2.1 Study design

**Figure 1:**
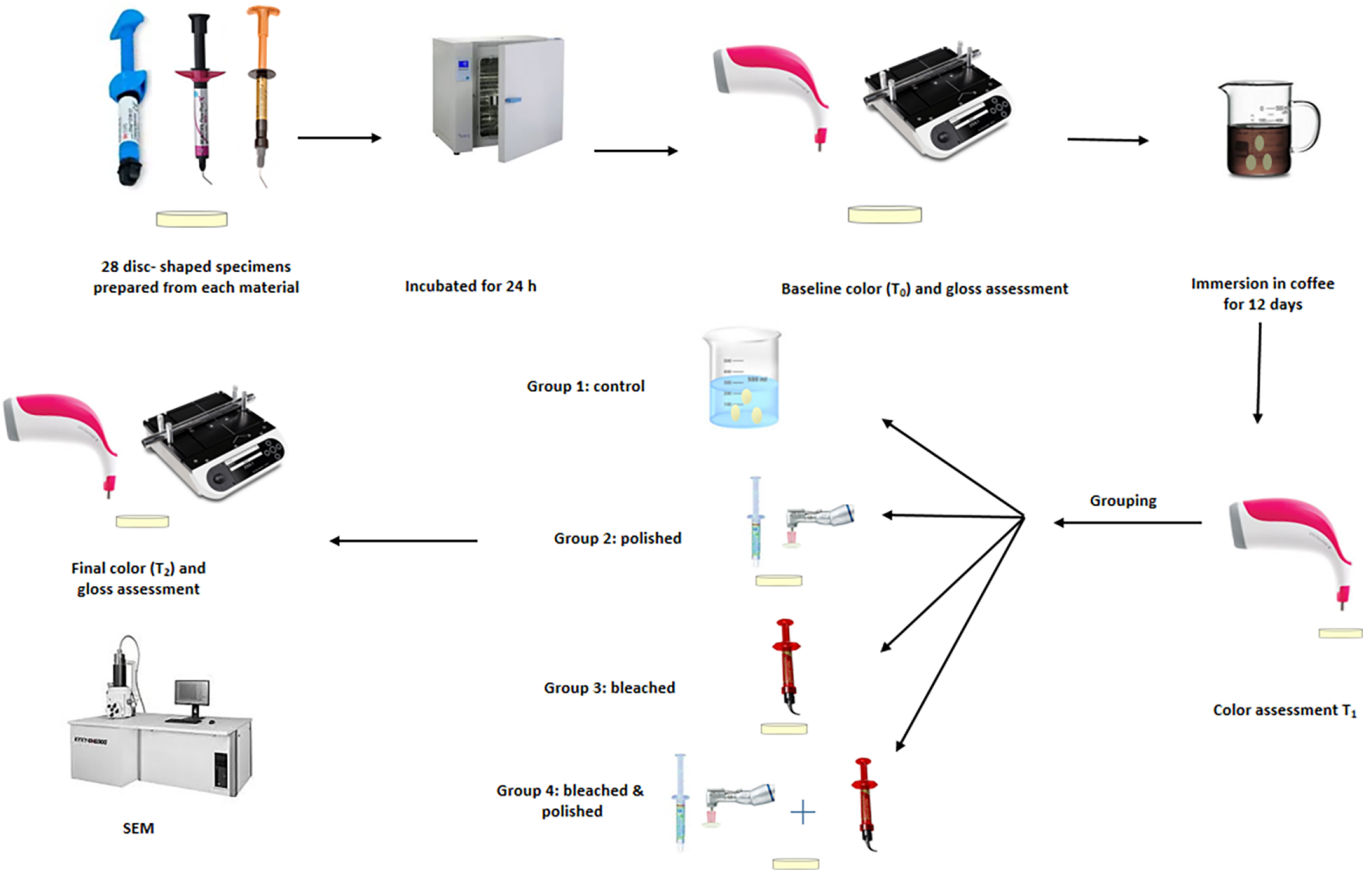
Graphical representation of the study design.

This investigation was performed after approval of the Research and Ethics Committee Ref. No: RAKMHSU-REC-8-2023/24-UG. The materials investigated are one sculptable nanofilled composite (Filtek™ Z350XT Universal Restorative, 3M ESPE, USA) and two injectable composite resins: Beautifil Flow Plus X (Shofu Inc, Kyoto, Japan) and G-ænial® Universal Injectable (GC Corp., USA), Details of the materials utilized in the current study are listed in (Table 1). The study design is illustrated in (Figure 1).

**Table 1:** The commercial brand names, compositions, and manufacturers of the materials used in the study.

Brand name	Material	Code	Composition	Manufacturer	Lot
I. The composite restorative materials					
Filtek™ Z350XT Universal Restorative.	Nanofilled composite restorative material shade: A2	FUR	**Matrix:** Bis-GMA, UDMA, Bis-EMA 6, and small quantities of TEGDMA. **Filler:** 78.5% Non-agglomerated nanoparticles of silica 20 nm size and nano-agglomerates formed of zirconium/silica particles ranging from 0.6 to 1.4 μm.	3M ESPE, St. Paul, MN, USA	10229114.
Beautifil Flow Plus X. F00.	Nanofilled injectable giomer, shade: A2	BFP	*Organic matrix:* 10%–20% Bis-GMA, TEGDMA, Bis-MPEPP, polymerization initiator, pigments, others. *Filler:* 50%–60% S-PRG fillers based on aluminofluoroborosilicate glass, Al2O3. Filler size: 0.8 μm.	Shofu Inc, Kyoto, Japan.	082263.
G-ænial® Universal Injectable.	Nanofilled injectable composite, shade: A2	GUI	**Matrix:** UDMA, bis-EMA, methacrylate monomers, photoinitiator, UV-light absorber, pigments. **Filler:** Barium (Ba) glass, Silicon dioxide (SiO2). Filler %: (wt%/vol%) 69/50. Wt%: 69%, size:150 nm	GC Corp., USA.	230519A.
II. The stain-removing methods					
Super-Snap®Buff Disk.	Polishing discs.		Synthetic felt-coated polishing disks	Shofu Inc., Kyoto, Japan.	PN 0536.
DirectDia Paste.	Polishing paste.		20% diamond particles in grain size 2–4 µm.	Shofu Inc., Kyoto, Japan.	0123234.
Opalescence® Boost PF 40%.	(office bleaching		40% hydrogen peroxide, potassium nitrate, and fluoride.	Ultradent Products Inc., South Jordan, UT.	BVD8Y.

Bis-GMA, bisphenol a-glycidyl methacrylate; UDMA, urethane dimethacrylate; Bis-EMA, bisphenol A ethoxylated dimethacrylate; TEGDMA, triethylene glycol dimethacrylate; Bis-MPEPP, bisphenol A polyethylene glycol polypropane diol dimethacrylate; S-PRG, surface pre-reacted glass; Al2O3, aluminum oxide; Ba, barium; SiO2, silicon dioxide; wt%, weight percent; vol%, volume percent; μm, micrometer; Nm, nanometer.

### 2.2 Sample size calculation

A priori sample size calculation was performed using the software G*Power 3.1.9.4 before starting the study. The minimum sample size determined was 84 for an effect size of 0.71 (21) at 95% power and 5% confidence interval.

### 2.3 Specimens preparation

Eighty-four disc-shaped specimens, twenty-eight of each restorative material, were prepared in a custom-made silicon mold (10 mm diameter and 2 mm thickness). The mold was positioned over a glass plate topped with a Mylar strip, and then the mold hole was filled with the composite resins. For the sculptable FUR composite, a plastic instrument was used to adapt one layer of the composite paste inside the mold hole, while BFP and GUI were directly injected without sculpting (20, 25). An additional Mylar strip was placed atop the composite, and gentle pressure (5–10 N) was applied using another glass plate until it was level with the mold's upper surface to smoothen the composite specimens and extrude any excess (25). Specimens underwent light curing for 20 s on each surface following their respective manufacturer's instructions, using a LED curing lamp (Elipar™ DeepCure-L 3M ESPE, St Paul, USA), with output 1,000 mW/cm^2^. The samples were subsequently kept in distilled water for 24 h at 37°C to ensure their complete polymerization.

### 2.4 The staining challenge of the specimens

The staining solution was prepared by dissolving 4 grams of instant coffee (DAVIDOFF Fine Aroma, Tchibo Manufacturing, Poland) into 200 ml of boiling water for 2 min and cooling to room temperature. The specimens were submerged in the staining solution and incubated for 12 days at 37°C, with the solution being replaced every 24 h to mimic one year of typical coffee intake. The specimens were washed with tap water before testing.

### 2.5 Staining susceptibility

Color measurements of each specimen were conducted using a portable spectrophotometer (VITA Easyshade® Advance 4.0, Vita Zahnfabrik, Bad Säckingen, Germany) following the CIELAB color space standard established by the Commission Internationale de l'Eclairage. After calibrating the device, the spectrophotometer probe was centrally placed on each specimen, positioned on a white non-reflective surface to eliminate the background interference, and illuminated under a D65 light source. The color parameters (L, C and H) for each specimen were recorded initially before staining (T_0_), and after the coffee staining challenge (T_1_). Then, the color change (staining susceptibility) ΔE_00_ (T_0_-T_1_) of each specimen was calculated according to the CIEDE2000 equation as follows (26):

$$\Delta E_{00}={\left[{\left(\frac{\Delta L^{\prime }}{K_{L}S_{L}}\right)}^{2}+{\left(\frac{\Delta C^{\prime }}{K_{C}S_{C}}\right)}^{2}+{\left(\frac{\Delta H^{\prime }}{K_{H}S_{H}}\right)}^{2}+R_{T}\left(\frac{\Delta C^{\prime }}{K_{C}S_{C}}\right)\left(\frac{\Delta H^{\prime }}{K_{H}S_{H}}\right)\right]}^{\frac{1}{2}}$$

Δ*L΄*, Δ*C΄*, and Δ*H΄* represent the variations in Lightness, Chroma, and Hue, respectively. The rotation function is *R*_*T*_, whereas *S*_*L*_*, S*_*C*_, and *S*_*H*_ are weighting functions; *K*_*L*_, *K*_*C*_, and *K*_*H*_ are parameters for experimental adjustment. In this study, these parametric variables were set to a default value of 1 (27). The staining susceptibility of the tested materials was further assessed according to the 50:50% perceptibility PT (0.80 and acceptability thresholds AT (1.8) established by Paravina et al. (28), and following the International Organization for Standardization guidelines (ISO/TR 28642:2016) (29).

### 2.6 Grouping of the specimens and stain removal assessment

After the immersion in the coffee solution, the specimens for each material were randomly divided into four subgroups, each of 7 specimens, as follows:

Group 1: Specimens received no stain removal treatment and were kept in distilled water (control group).

Group 2: Specimens were polished (Super-Snap Buff Polisher + Direct DiaPaste) for 60 s at a contact pressure of 0.5 N (50 g) load in a clockwise rotation motion using a low-speed handpiece speed 5,000 rpm, as recommended by the manufacturer. The pressure was controlled using a precision scale.

Group 3: Specimens were subjected to in-office bleaching treatment (Opalescence Boost 40% hydrogen peroxide gel), applied for one hour (3 rounds/20 min each) as recommended by the manufacturer.

Group 4: The specimens were subjected to a combination of in-office bleaching (as in group 3) followed by polishing (as in group 2).

A single operator performed the bleaching and polishing procedures to reduce possible variability. Color parameters (L, C and H) for each specimen were recorded again after the different stain removing methods T_2_ according to the method described above and the color change was calculated according to the CIEDE2000 equation ΔE_00_ (T_0_-T_2_).

### 2.7 Surface gloss test

Gloss assessments were conducted using a small area gloss meter (Novo-Curve, Rhopoint Instrumentation Ltd., UK) at a 60° angle for both light reflection and incidence as recommended by the International Organization for Standardization standard for intermediate gloss materials (ISO 2813:2014) (30). Each specimen was positioned over a 2 mm × 2 mm measurement area and obscured by a black shield to mitigate external light interference during the measurement process. Before testing, the apparatus was calibrated using a calibration plate according to the manufacturer's instructions. Gloss values were measured in gloss units (GU). A highly polished surface with a refractive index of 1.567 achieves a value of 100 GU, whereas a non-reflective surface is assigned a value of zero (0 GU). Three measurements were taken for each specimen and averaged to determine its respective gloss value. The gloss was measured initially upon specimen preparation and after the different stain removing methods.

### 2.8 Scanning electron microscopy

One specimen from each group was randomly selected, and one baseline untreated specimen from each material were gold plated then fixed on unique aluminum studs to examine the surface morphology under Scanning Electron Microscopy (Model FEI Quanta 3D 200i, FEI Company) at 2,000× magnification.

### 2.9 Statistical analysis

Means and standard deviations were calculated for each group. The data was normally distributed after performing the Kolmogorov–Smirnov and Shapiro–Wilk test. A one-way analysis of variance (ANOVA) was performed to assess the staining susceptibility of the three materials, while color change after stain removal and surface gloss results were analyzed using a two-way ANOVA to evaluate the effects of the independent variables: different materials and methods of stain removal. When significant differences were detected, Tukey's HSD *post hoc* test was performed for multiple comparisons between the groups. The level of significance was set at *P* < 0.05. Data analysis was conducted using SPSS® version 27 (SPSS**®** Inc., IBM Corp., New York, USA).

## 3 Results

### 3.1 Color change results

**Figure 2:**
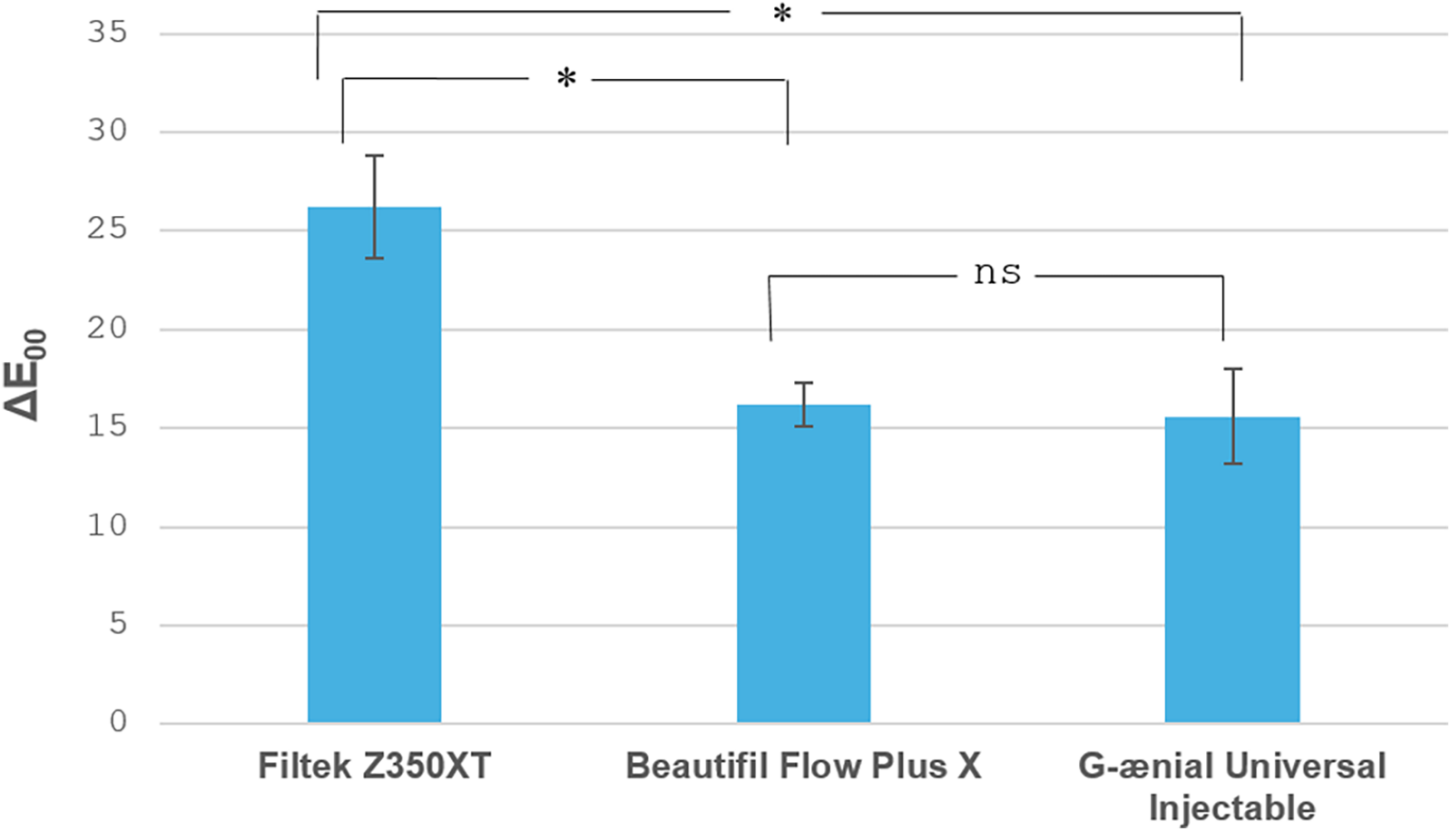
Bar chart displaying the staining susceptibility (∆E_00_) of the tested restorative materials. * indicates significant difference, while ns indicates non significant difference at *P* < 0.05.

**Figure 3:**
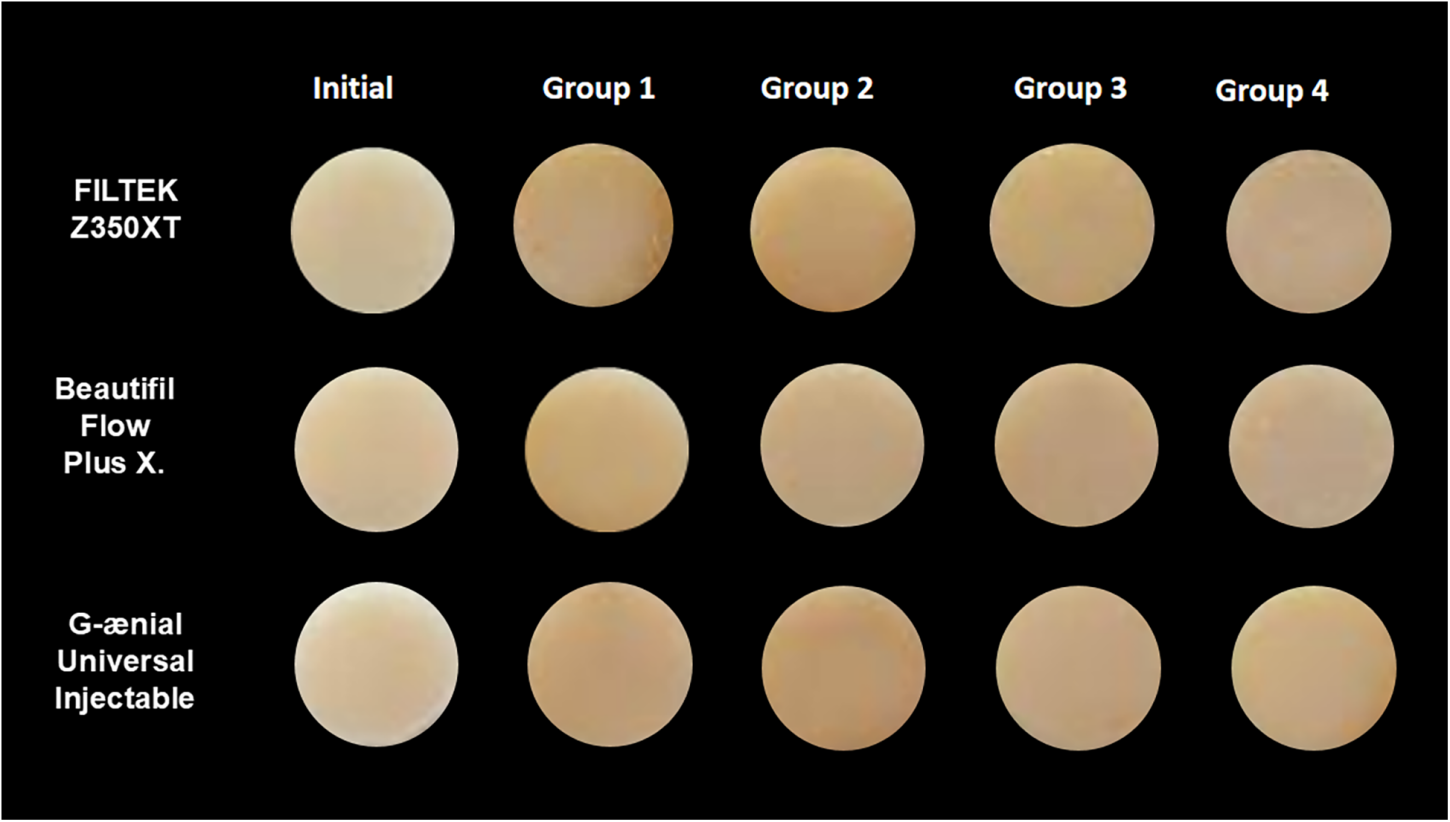
Representative specimens color change during the study.

Overall, all tested materials exhibited a clinically unacceptable color change, exceeding the acceptability threshold (AT) after immersion in coffee and following stain removal methods. The staining susceptibility results are shown in (Figure 2). One-way ANOVA revealed a statistically significant difference among the groups (*P* < 0.001), the highest color change was recorded for FUR which was significantly higher than BPF & GUI (*P* < 0.05), with no significant difference between BPF and GUI in the same group (*P* > 0.05). Comparing the stain removal methods on the three materials to their control groups, two-way ANOVA showed significant interaction between the variables (*P* < 0.001). FUR showed a significant reduction in color change in group 2 (*P* < 0.05), with a further significant reduction in the ΔE**_00_** values in groups 3 and 4 (*P* < 0.05), with no significant difference between them (*P* > 0.05). However, the BPF and GUI color change values were statistically similar in groups 2, 3, and 4 (*P* > 0.05), and all showed a significant reduction in color change values compared to their respective control group (*P* < 0.05) (Table 2 and Figure 3).

**Table 2:** The color change (ΔE_00_) means and (standard deviations) of the tested restorative materials.

Material/groups	Group 1 control	Group 2 polished	Group 3 bleached	Group 4 bleached & polished
Filtek Z350 XT (FUR).	26.2 (2.6)^Aa^	19.2 (3.8)^Ab^	16.0 (1.6)^Ac^	14.3 (1.6)^Ac^
Beautifil Flow Plus X (BFP).	16.1 (1.1)^Ba^	11.2 (1.9)^Bb^	10.3 (1.4)^Bb^	10.3 (2.3)^Bb^
G-aenial Universal Injectable (GUI).	15.6 (2.4)^Ba^	12.4 (4.3)^Bb^	11.8 (2.6)^Bb^	10.4 (2.6)^Bb^

Different letters within columns and lines indicate statistically significant differences (*p* < 0.05). Lowercases represent linear differences, while uppercases represent columnar differences.

### 3.2 Surface gloss results

Means and standard deviations for the surface gloss of the tested materials are shown in (Table 3). Two-way ANOVA revealed a significant interaction between the variables (*P* < 0.001). Multiple comparisons revealed no significant change in the gloss values between baseline readings, group 1 and group 3 in the three materials (*P* > 0.05). In contrast, a significant decrease was recorded in all the materials in groups 2 and 4 (*P* < 0.05). FUR gloss values were significantly reduced in group 2, and further significant reduction was recorded in group 4 (*P* < 0.05). BFP had a significant reduction in gloss values in groups 2 and 4 with no significant difference between them. GUI had a significant reduction in gloss values in group 4, followed by a further significant reduction in group 2 (*P* < 0.05).

**Table 3:** The surface gloss (GU) means and (standard deviations) of the tested restorative materials.

Material/groups	Baseline	Group 1 control	Group 2 polished	Group 3 bleached	Group 4 bleached & polished
Filtek Z350XT (FUR).	51.7 (6.6)^Aa^	52.8 (11.2)^Aa^	31.6 (5.7)^Ab^	50.3 (11.9)^Aa^	22.3 (11.1)^Ac^
Beautifil Flow Plus X (BFP).	51.1 (12.2)^Aa^	49.7 (9.4)^Aa^	17.9 (3.7)^Bb^	47.8 (8)^Aa^	19 (2.3)^Ab^
G-aenial universal injectable (GUI).	52.4 (9.6)^Aa^	51.4 (9.7)^Aa^	15.4 (1.5)^Bb^	50 (8.3)^Aa^	23.9 (7.3)^Ac^

Different letters within columns and lines indicate statistically significant differences (*p* < 0.05). Lowercases represent linear differences, while uppercases represent columnar differences.

### 3.3 Scanning electron microscope results

**Figure 4:**
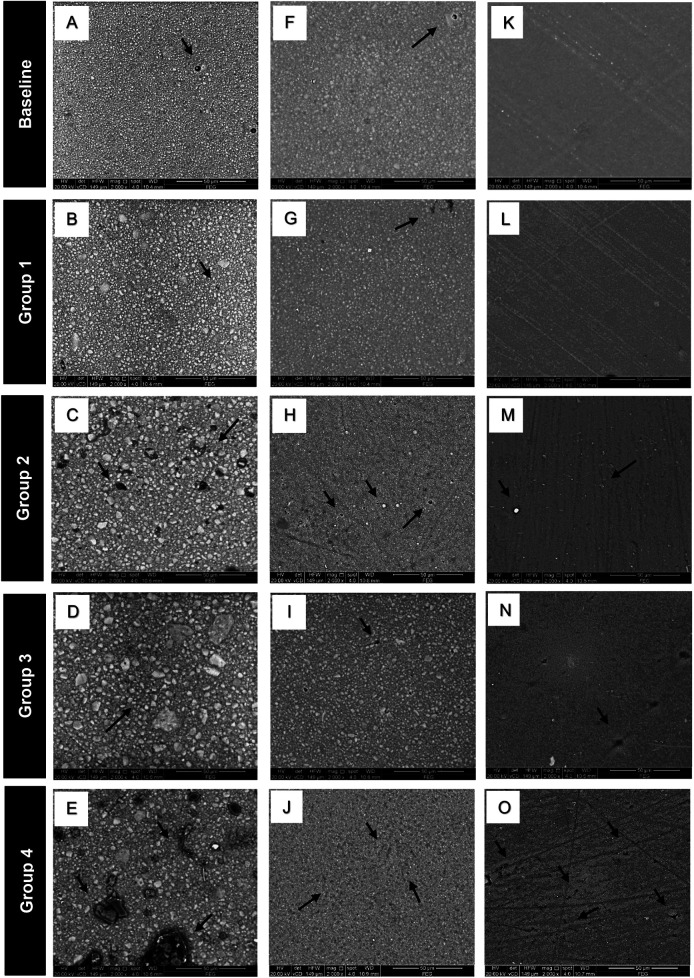
Representative SEM images of the tested materials. Filtek Z350XT **(A–E)**: Baseline **(A)** shows a smooth surface with few voids. Group 1 **(B)** maintains a smooth surface with minimal voids. Group 2 **(C)** exhibits filler dislodgment and multiple wide voids. Group 3 **(D)** shows fewer surface defects compared to Group 2. Group 4 **(E)** shows larger areas of filler dislodgment and extensive void formation. Beautifil Flow Plus X **(F–J)**: Baseline **(F)** and Group 1 **(G)** display smooth surfaces with few voids. Group 2 **(H)** has a slightly higher number of small voids compared to Group 3 **(I)**, while Group 4 **(J)** exhibits multiple small voids and noticeable filler dislodgment. G-aenial Universal Injectable **(K–O)**: Baseline **(K)** and Group 1 **(L)** exhibit the smoothest, most intact surfaces. Groups 2 **(M)** and 3 **(N)** show a few small voids. Group 4 **(O)** presents multiple small voids and noticeable filler dislodgment.

The SEM images of the tested materials are presented in Figure 4. Baseline images (A, F and K) and group 1 (B, G and L) showed smoother surfaces among all the groups with minimal voids detected in FUR (A and B) and BPF (F and G), while the GUI samples showed the smoothest intact surfaces both at baseline (K) and in group 1. Group 2 showed dislodgment of fillers which was more evident in FUR (C), and to a lesser extent in BPF and GUI (H and M). Group 3 showed less surface defects (D, I and N) than group 2. The most significant surface defects were detected in group 4 with larger areas of filler dislodgment and huge voids in FUR (E), and a greater amount of filler loss with subsequent multiple small voids in BPF and GUI (J and O) respectively.

## 4 Discussion

The current study investigated the staining potential of two new injectable composite resins compared to conventional paste-like sculptable nanofilled composite resin and the stain removal ability of polishing, bleaching, or their combination on these materials. Based on the results of the present *in vitro* study, both the null hypothesis were rejected.

Dental restorations are exposed to various staining beverages during clinical practice. The choice to submerge the test specimens in coffee stemmed from its widespread everyday use globally. Accelerated aging was performed through immersion of the specimens in the staining medium for twelve days, equivalent to nearly one year of intraoral exposure as reported in earlier studies (31, 32). While coffee is consumed worldwide as both a hot and cold beverage, the coffee solution in the current study was utilized at room temperature, consistent with prior research, to eliminate temperature as a variable that could influence the results and to limit the impact of coffee on its pronounced chromogenic effect (16, 19).

The CIEDE2000 (ΔE_00_) formula was utilized to calculate the color difference, as it more accurately reflects human perceptions of color variation compared to the CIELAB formula (33). Besides the statistical analysis, the color stability results in this study were further assessed against the 50:50% acceptability threshold (AT) and the perceptibility threshold (PT) in CIEDE2000, which states that ΔE_00_ values less than or equal to 0.8 signify that color change is undetectable to the human eye (AT) whereas ΔE_00_ values less than 1.8 are perceptible yet clinically acceptable (PT) (28).

In the current study, all materials subjected to immersion in coffee as well as various stain removal procedures exhibited ΔE**_00_** values exceeding the established acceptability threshold. Consequently, these color alterations are deemed clinically inappropriate for those who drink coffee daily, necessitating restorative replacement after an interval of one year (11). This aligns with previous studies that deemed coffee a potent discolorant for dental resin-based restorations (16, 27, 34). Coffee comprises a variety of poly-phenolic compounds that exhibit health-enhancing properties for humans, including antioxidant and neuroprotective effects (35). However, composite resin materials can be drastically penetrated by the less polar and water-soluble polyphenols such as caffeine, tannin, and chlorogenic acid found in coffee (16). The highest staining susceptibility occurred in the conventional nanofilled composite FUR, which was significantly higher than both injectable composite materials, in line with previous studies (36, 37). In a study conducted by Nasim, et al. (38), Filtek Z350 exhibited the greatest degree discoloration in coffee solution compared to microhybrid and microfilled composites, they asserted that the cause might be related to the characteristics of the resin matrix and the porosity of the glass fillers nanoclusters. Besides, Cinelli, et al. (24), suggested a higher staining susceptibility in composites containing nano-aggregated particles as these structures possess an interface that is not fully silanized, which may result in increased penetration of water and pigments.

A noteworthy finding in the current investigation is that both the injectable composites showed reduced staining susceptibility in coffee solution, excelling the nanofilled paste composite, which has a greater filler content. Besides, both BPF and GUI had similar color change values in the untreated control group despite their different matrix composition; this indicates that additional factors influence the performance of these injectable composites. While no existing studies have examined the staining potential of injectable composites for direct comparison with the current study's results, one possible explanation lies in the employed technology, which ensures dense packing of smaller filler particles or enhanced silane bonding between fillers and the organic matrix are responsible for reduced staining potential (7, 32). Another explanation is that the tested nanofilled composite is paste-like sculptable material, with a higher likelihood of air bubble entrapment within the composite during their application and sculpting. In contrast, both the injectable composites have flowable consistency and were directly injected into the mold through the specially designed plungers without sculpting, thus reducing the possibility of air bubble entrapment (39). The water absorption capacity of composite materials is enhanced by the presence of porosity, which in turn leads to the accumulation of stains (40).

When evaluating the impact of stain-removing methods on teeth and associated restorations, it is crucial to distinguish between superficial stains and intrinsic discoloration (18). Polishing with Super-Snap Buff Disk and DirectDia Paste was chosen in the current investigation as it can effectively remove surface stains and restore gloss to the composite surface according to the manufacturer; besides, Szczepaniak, et al, reported its effectiveness as a polishing system without affecting the surface roughness of resin composites (41). On the other hand, in-office bleaching with 40% hydrogen peroxide offers a potent chemical approach to eliminating surface and deeper discoloration. It has been demonstrated to effectively remove stains from composite resins, frequently restoring them to their baseline color (11). The combination of the two methods was also tested as it was expected to offer a more comprehensive stain removal than either method alone (21).

In the present study, all the stain-removing methods were effective in partially eliminating the coffee stains of the tested materials, all the ΔE_00_ values were beyond the acceptability threshold, and their effect was material-dependent. Both bleaching (group 3) and bleaching followed by polishing (group 4) showed the greatest stain-removing efficacy for FUR more than polishing alone in group 2; this may indicate a deeper penetration of the stains beyond the surface layer, this is in agreement with Turkun and Turkun, who found that polishing was less effective than 15% hydrogen peroxide bleaching. However, it eradicated a portion of the stain. Cinelli et al., reported in their study on pigment penetration analysis of composite resin that the pigments can penetrate up to 1 mm depth in nanofilled composites and to 2 mm in micro-hybrid composites, which in either case cannot be eliminated by surface polishing alone (18, 24).

On the other hand, the polishing, bleaching, and bleaching followed by polishing showed a similar stain elimination pattern in both tested injectable composites BPF and GUI, which highly suggests that a superficial staining pattern occurred in these composites, the association between discoloration and water sorption of resin composites can justify this finding (42). The GUI matrix comprises Bis-EMA and UDMA monomers, which exhibit reduced water sorption levels of 20.1 and 29.5 μg/mm^3^, respectively. Moreover, The dispersed nanosized filler particles, which are securely integrated into the resin matrix via Full-coverage Silane Coating (FSC) technology, likely ensure a stable and robust matrix-filler bond that can substantially withstand the penetration by the acidic coffee pigmentation (43). Nevertheless, although earlier generations of Beautifil Flow Plus flowable giomer showed high water sorption values that increased after four weeks to reach up to 32.2 μg/mm3 (44, 45), the recently released generation utilized in the current study was reported in a recent study by Rusnac, et al., to have reduced water sorption of 15.4 μg/mm3 after 30 days of immersion in distilled water (46).

The gloss parameter, which mimics the natural appearance of teeth, significantly impacts the success rates of aesthetic restorations, alongside their color stability. It is an optical characteristic determined by how intensely light is reflected. Several factors influence gloss, including the angle at which light hits the surface, the refractive index of the material's components, and its surface characteristics (22). The present study utilized a 60° angle of light incidence, as advised by the ISO 2813:2014 standards for intermediate gloss materials (30). Thus, the gloss measurements depended on the surface topography and the material's refractive index. When a surface is irregular, it tends to scatter light rather than reflect it, resulting in lower gloss values, which can severely impact the aesthetics of resin composites and create disharmony between the restored and surrounding teeth. While there is no definitive standard for gloss values in dental composites, it is generally recommended to maintain gloss values between 40 and 60 GU (Gloss Units) (47).

In the current investigation, all the materials showed a similar gloss behavior, that they had clinically accepted gloss values initially, in stained untreated group 1 and after bleaching in group 3. However, the gloss values reduced to be clinically unaccepted after polishing in group 2 and bleached followed by polished group 4; the later groups showed evident surface defects in SEM images. Previous studies reported that coffee as well as hydrogen peroxide bleaching reduces the microhardness of composite resins (12, 48). The acidic nature of coffee can cause hydrolysis of the ester groups in the resin matrix, compromising its structure. This chemical breakdown can induce the weakening of the resin matrix, resulting in lower surface hardness. This decrease in surface hardness rendered the material more susceptible to wear from external pressures such as polishing in the current study (49). Furthermore, the hydrogen peroxide in the bleaching agent promotes the formation of free radicals through the oxidation process; these free radicals can cause hydrolytic degradation of composite resin at the resin-filler interface, aiding filler-matrix de-bonding once subjected to the external wear mechanism by repolishing after bleaching, with subsequent reduction in their gloss values (50, 51).

While accelerated aging for twelve days in the coffee solution in the current investigation aims to predict long-term material performance, the expedited nature of this process may not accurately represent the natural aging of materials over extended periods, such as months or years, in clinical applications. The present investigation findings are also limited to laboratory conditions, in which some oral environment factors were not fully replicated, such as intraoral temperature fluctuations, the effect of other beverages, and saliva's buffering effect. Further studies should consider increasing the frequency of stain removal methods and studying the translucency and opalescence properties of the injectable composites.

## 5 Conclusions

Considering the current study's limitations, the staining susceptibility of Beautifil Flow Plus X and G-ænial Universal Injectable composites was less than Filtek™ Z350XT Universal Restorative composite. None of the stain-removing methods could restore the baseline color of all the composites. In-office bleaching with Opalescence Boost 40% effectively maintained an optimal surface gloss. Polishing alone or after bleaching is not recommended to eliminate resin composite coffee stains due to their gloss reduction effect.

## Data Availability

The original contributions presented in the study are included in the article/Supplementary Material, further inquiries can be directed to the corresponding author.

## References

[1] Kouri V Moldovani D Papazoglou E. Accuracy of direct composite veneers via injectable resin composite and silicone matrices in comparison to diagnostic wax-up. J Funct Biomater. (2023) 14(1):32. 10.3390/jfb14010032 36662079 PMC9864032

[2] da Cunha LF Reis R Santana L Romanini JC Carvalho RM Furuse AY. Ceramic veneers with minimum preparation. Eur J Dent. (2013) 7(04):492–6. 10.4103/1305-7456.120645 24932126 PMC4053676

[3] Jr N F Ritter AV. Composite veneers: the direct–indirect technique revisited. J Esthet Restor Dent. (2021) 33(1):7–19. 10.1111/jerd.12696 33336852

[4] Al Ghamdi Z. Layering technique of resin composite method for direct anterior teeth restorations: a new appraisal. Saudi J Oral Dent Res. (2023) 8(7):219–22. 10.36348/sjodr.2023.v08i07.003

[5] Geštakovski D. The injectable composite resin technique: biocopy of a natural tooth–advantages of digital planning. Int J Esthet Dent. (2021) 16(3):280–99. 34319664

[6] Coachman C De Arbeloa L Mahn G Sulaiman T Mahn E. An improved direct injection technique with flowable composites. A digital workflow case report. Oper Dent. (2020) 45(3):235–42. 10.2341/18-151-T 32101498

[7] Elsahn NA El-Damanhoury HM Shirazi Z Saleh ARM. Surface properties and wear resistance of injectable and CAD/CAM–milled resin composite thin occlusal veneers. Eur J Dent. (2023) 17(03):663–72. 10.1055/s-0042-1750769 36220115 PMC10569885

[8] Degirmenci A Degirmenci B Salameh M. Long-term effect of acidic beverages on dental injectable composite resin: microhardness, surface roughness, elastic modulus, and flexural strength patterns. Strength Mater. (2022) 54(2):331–43. 10.1007/s11223-022-00409-z

[9] Rajabi H Denny M Karagiannopoulos K Petridis H. Comparison of flexural strength and wear of injectable, flowable and paste composite resins. Materials (Basel). (2024) 17(19):4749. 10.3390/ma17194749 39410319 PMC11477787

[10] Gia NR Y Sampaio CS Higashi C Sakamoto Jr A Hirata R. The injectable resin composite restorative technique: a case report. J Esthet Restor Dent. (2021) 33(3):404–14. 10.1111/jerd.12650 32918395

[11] Alharbi A Ardu S Bortolotto T Krejci I. In-office bleaching efficacy on stain removal from CAD/CAM and direct resin composite materials. J Esthet Restor Dent. (2018) 30(1):51–8. 10.1111/jerd.12344 29130615

[12] El-Sayed HY Abdalla AI El-Ebiary MA El-Eraky M Farghal NA. The effects of two bleaching agents on the physico-mechanical properties of three resin-based restorative materials. Int J Clin Dent. (2009) 2(2):69–86.

[13] Akgul N Yilmaz MN. Translucency and contrast ratio of dimethacrylate resin-based dental materials after bleaching: an *in vitro* study. BMC Oral Health. (2024) 24:1564. 10.1186/s12903-024-05383-3 39731149 PMC11681738

[14] Matzinger M Hahnel S Preis V Rosentritt M. Polishing effects and wear performance of chairside CAD/CAM materials. Clin Oral Investig. (2019) 23:725–37. 10.1007/s00784-018-2473-3 29770877

[15] Farah RI Elwi H. Spectrophotometric evaluation of color changes of bleach-shade resin-based composites after staining and bleaching. J Contemp Dent Pract. (2014) 15(5):587–94. 10.5005/jp-journals-10024-1584 25707831

[16] Hashemikamangar SS Farahani S Khoshgoo S Doroudgar P. Comparative efficacy of four stain removal methods for bleach-shade composite resins after immersion in staining solutions: an *in vitro* study. Int J Dent. (2023) 2023:1–7. 10.1155/2023/8909288 37342250 PMC10277192

[17] Villalta P Lu H Okte Z Garcia-Godoy F Powers JM. Effects of staining and bleaching on color change of dental composite resins. J Prosthet Dent. (2006) 95(2):137–42. 10.1016/j.prosdent.2005.11.019 16473088

[18] LŞ T Türkün M. Effect of bleaching and repolishing procedures on coffee and tea stain removal from three anterior composite veneering materials. J Esthet Restor Dent. (2004) 16(5):290–301. 10.1111/j.1708-8240.2004.tb00056.x 15726798

[19] Korać S Ajanović M Džanković A Konjhodžić A Hasić-Branković L Gavranović-Glamoč A Color stability of dental composites after immersion in beverages and performed whitening procedures. Acta Stomatologica Croatica: Inter J of Oral sci Dent Med. (2022) 56(1):22–32. 10.15644/asc56/1/3 PMC8972476 35382484

[20] Korkut B Hacıalı C. Color stability of flowable composites in different viscosities. Clin Exp Health Sci. (2020) 10(4):454–61. 10.33808/clinexphealthsci.816231

[21] Rodrigues C Nora BD Mallmann A May L Jacques L. Repolishing resin composites after bleaching treatments: effects on color stability and smoothness. Oper Dent. (2019) 44(1):54–64. 10.2341/17-107-L 29856701

[22] Amaya-Pajares SP Koi K Watanabe H da Costa JB Ferracane JL. Development and maintenance of surface gloss of dental composites after polishing and brushing: review of the literature. J Esthet Restor Dent. (2022) 34(1):15–41. 10.1111/jerd.12875 35088935

[23] Farghal NS Awadalkreem F Abouelhonoud NA Khan RI. The gloss retention of esthetic restorations following simulated brushing with charcoal oral products: an *in vitro* study. J Contemp Dent Pract. (2024) 25(5):473–9. 10.5005/jp-journals-10024-3692 39364847

[24] Cinelli F Scaminaci Russo D Nieri M Giachetti L. Stain susceptibility of composite resins: pigment penetration analysis. Materials (Basel). (2022) 15(14):4874. 10.3390/ma15144874 35888342 PMC9320780

[25] Uctasli M Garoushi S Uctasli M Vallittu P Lassila L. A comparative assessment of color stability among various commercial resin composites. BMC Oral Health. (2023) 23(1):789. 10.1186/s12903-023-03515-9 37875872 PMC10598901

[26] Sharma G Wu W Dalal EN. The CIEDE2000 color-difference formula: implementation notes, supplementary test data, and mathematical observations. Color Res Appl. (2005) 30(1):21–30. 10.1002/col.20070

[27] Farghal NS Sheikh Debis MN Abou Baker T Mahmoud O. The influence of the new charcoal toothbrush and toothpaste on esthetic restoration properties. Int J Dent. (2024) 2024(1):4385524. 10.1155/2024/4385524

[28] Paravina RD Pérez MM Ghinea R. Acceptability and perceptibility thresholds in dentistry: a comprehensive review of clinical and research applications. J Esthet Restor Dent. (2019) 31(2):103–12. 10.1111/jerd.12465 30891913

[29] ISO/TR 28642. Dentistry-Guidance on Colour Measurement. Geneva: International Standards Organization (ISO) (2016). p. 1–10.

[30] ISO 2813:2014. Paints and Varnishes—Determination of Gloss Value at 20°, 60° and 85°. Geneva: International Organization for Standardization (2014).

[31] Alharbi N Alharbi A Osman R. Stain susceptibility of 3D-printed nanohybrid composite restorative material and the efficacy of different stain removal techniques: an *in vitro* study. Materials (Basel). (2021) 14(19):5621. 10.3390/ma14195621 34640015 PMC8510074

[32] Jrady A Ragab H Algahtani FN Osman E. *In vitro* study on the impact of various polishing systems and coffee staining on the color stability of bleach-shaded resin composite. BMC Oral Health. (2024) 24(1):712. 10.1186/s12903-024-04474-5 38902697 PMC11191283

[33] Gómez-Polo C Muñoz MP Luengo MCL Vicente P Galindo P Casado AMM. Comparison of the CIELab and CIEDE2000 color difference formulas. J Prosthet Dent. (2016) 115(1):65–70. 10.1016/j.prosdent.2015.07.001 26412001

[34] Kim S Lee C-H Ma S Park Y-S. Whitening efficacy of toothpastes on coffee-stained teeth: an enamel surface analysis. Int Dent J. (2024) 74(6):1233–8. 38614882 10.1016/j.identj.2024.02.006 PMC11551553

[35] Bukowska B LIP. Tea and coffee polyphenols and their biological properties based on the latest *in vitro* investigations. Ind Crops Prod. (2022) 175:114265. 10.1016/j.indcrop.2021.114265 34815622 PMC8601035

[36] Da Costa JB Goncalves F Ferracane JL. Comparison of two-step versus four-step composite finishing/polishing disc systems: evaluation of a new two-step composite polishing disc system. Oper Dent. (2011) 36(2):205–12. 10.2341/10-162-L 21702670

[37] Vulović S Stašić JN Ilić J Todorović M Jevremović D Milić-Lemić A. Effect of different finishing and polishing procedures on surface roughness and microbial adhesion on highly-filled composites for injectable mold technique. J Esthet Restor Dent. (2023) 35(6):917–26. 10.1111/jerd.13045 37039335

[38] Nasim I Neelakantan P Sujeer R Subbarao C. Color stability of microfilled, microhybrid and nanocomposite resins—an *in vitro* study. J Dent. (2010) 38:e137–e42. 10.1016/j.jdent.2010.05.020 20553993

[39] Gjorgievska E Oh DS Haam D Gabric D Coleman NJ. Evaluation of efficiency of polymerization, surface roughness, porosity and adaptation of flowable and sculptable bulk fill composite resins. Molecules. (2021) 26(17):5202. 10.3390/molecules26175202 34500635 PMC8434499

[40] Sarna-Boś K Skic K Sobieszczański J Boguta P Chałas R. Contemporary approach to the porosity of dental materials and methods of its measurement. Int J Mol Sci. (2021) 22(16):8903. 10.3390/ijms22168903 34445606 PMC8396236

[41] Szczepaniak ME Krasowski M Bołtacz-Rzepkowska E. The effect of various polishing systems on the surface roughness of two resin composites—an *in vitro* study. Coatings. (2022) 12(7):916. 10.3390/coatings12070916

[42] Arregui M Giner L Ferrari M Vallés M Mercadé M. Six-month color change and water sorption of 9 new-generation flowable composites in 6 staining solutions. Braz Oral Res. (2016) 30(1):e123. 10.1590/1807-3107bor-2016.vol30.0123 27901205

[43] Bai X Chen Y Zhou T Pow EHN Tsoi JKH. The chemical and optical stability evaluation of injectable restorative materials under wet challenge. J Dent. (2024) 146:105031. 10.1016/j.jdent.2024.105031 38710315

[44] Harhash AY ElSayad II Zaghloul AG. A comparative *in vitro* study on fluoride release and water sorption of different flowable esthetic restorative materials. Eur J Dent. (2017) 11(02):174–9. 10.4103/ejd.ejd_228_16 28729788 PMC5502560

[45] Sokolowski K Szczesio-Wlodarczyk A Bociong K Krasowski M Fronczek-Wojciechowska M Domarecka M Contraction and hygroscopic expansion stress of dental ion-releasing polymeric materials. Polymers (Basel). (2018) 10(10):1093. 10.3390/polym10101093 30961019 PMC6403603

[46] Rusnac ME Prodan D Cuc S Petean I Prejmerean C Gasparik C Water sorption and solubility of flowable giomers. Materials (Basel). (2021) 14(9):2399. 10.3390/ma14092399 34063032 PMC8124910

[47] da Costa JB Ferracane JL Amaya-Pajares S Pfefferkorn F. Visually acceptable gloss threshold for resin composite and polishing systems. J Am Dent Assoc. (2021) 152(5):385–92. 10.1016/j.adaj.2020.09.027 33549304

[48] Barve D Dave PN Gulve MN Sahib MAM Naz F Shahabe SA. Effect of commonly consumed beverages on microhardness of two types of composites. Int J Clin Pediatr Dent. (2020) 13(6):663. 10.5005/jp-journals-10005-1854 33976493 PMC8060943

[49] Das K Murthy CS Naganath M Mehta D Anitha Kumari R Karobari MI Insights into the effects and implications of acidic beverages on resin composite materials in dental restorations: an *in vitro* study. J Esthet Restor Dent. (2024). 39614770 10.1111/jerd.13372

[50] Mohammadi N Alavi FN Rikhtehgaran S Chaharom MEE Salari A Kimyai S Effect of bleaching method and curing time on the surface microhardness of microhybrid composite resin. Maedica (Buchar). (2020) 15(3):359. 10.26574/maedica.2020.15.3.359 PMC7726494 33312252

[51] Chakraborty A Purayil T Ginjupalli K Pentapati KC Shenoy N. Effect of in-office bleaching agent on the surface roughness and microhardness of nanofilled and nanohybrid composite resins. F1000Res. (2023) 12:129. 37396049 10.12688/f1000research.130071.2 PMC10311121

